# High-Ranked Social Science Journal Articles Can Be Identified from Early Citation Information

**DOI:** 10.1371/journal.pone.0112520

**Published:** 2014-11-12

**Authors:** David I. Stern

**Affiliations:** Crawford School of Public Policy, The Australian National University, Acton, Australian Capital Territory, Australia; Max Planck Society, Germany

## Abstract

Do citations accumulate too slowly in the social sciences to be used to assess the quality of recent articles? I investigate whether this is the case using citation data for all articles in economics and political science published in 2006 and indexed in the *Web of Science*. I find that citations in the first two years after publication explain more than half of the variation in cumulative citations received over a longer period. Journal impact factors improve the correlation between the predicted and actual future ranks of journal articles when using citation data from 2006 alone but the effect declines sharply thereafter. Finally, more than half of the papers in the top 20% in 2012 were already in the top 20% in the year of publication (2006).

## Introduction

I show that citations received by journal articles in the social sciences in the first few years after publication are predictive for citations received in future years. This finding is of interest because it is usually assumed that citations accumulate too slowly in social sciences other than psychology to be useful for short-term research assessment [1]. For example, the Australian Government's Excellence in Research for Australia (ERA) exercise, which attempts to assess the research quality of universities in the previous 5 years, uses peer review in social science disciplines apart from psychology for this reason but uses citation analysis for psychology and all natural sciences. On the other hand, the Research Evaluation Framework (REF) in the United Kingdom uses peer review for all disciplines. Research evaluation exercises in other countries use different combinations of peer review and bibliometric analysis. For example, the Italian Evaluation of Research Quality must peer review at least half the submitted research items [2]. If it is not much more difficult to predict citations in social science disciplines than in natural science disciplines, then it should be possible to expand bibliometric analysis in such evaluation exercises to all disciplines apart from the humanities and arts.

There is an alternative to peer review and citation counting - using journal metrics such as the journal impact factor, which is widely used in many disciplines including economics to assess the potential quality of recently published papers [3]. However, correlations between impact factors and the citations received by individual articles in the respective journals are low [4] and use of impact factors for this purpose has been much criticized [5]. Hegarty and Walton show that article page length and reference list length are better predictors of citations to an individual article than the journal impact factor [6]. On the other hand, Bertocchi *et al*. show that, at least in economics, there is a strong correlation between peer review assessment of an article's quality and the impact factor of the journal in which it was published [2].

In this article, I use simple methods to test how well initial citations and journal impact factors can predict the future citations rankings of journal articles. I apply these methods to all journal articles included in the economics and political science categories in the *Web of Science* in 2006, tracking cumulative citations through 2012. These two fields represent a field where journal articles reign supreme (economics) and a field where books are also important (political science).

The absolute number of citations received by articles is much less important for evaluation purposes than determining which articles rank high or low. Therefore, I compute the rank correlation between cumulative citations from 2006 to 2012 and the partial sums of citations for earlier years. Obviously, as citations accumulate, the rank correlation will increase, but how fast the correlation rises is of interest. As there is particular interest in whether we can predict which articles will be in the top quality categories, I also compute how many articles that were in various top quantiles in 2012 were already in those quantiles in earlier periods. Finally, I test whether adding information about the journal in which an article is published can help predict its future rank. Sgroi and Oswald suggest that though the impact factor is a very imperfect predictor of individual article citations it can serve in a similar fashion to a Bayesian prior before citation data arrives [7]. Therefore, I estimate a series of simple regressions using the number of citations accumulated in a given initial period and impact factors to predict cumulative citations over the entire seven-year period. As suggested by Laband, the regression coefficient of the impact factor should decline as the initial period is extended [8]. I test the predictive quality of these models by computing the rank correlation of their predicted citations and actual cumulative citations.

The results show that using just citation data from the year of publication and the following year can explain more than half the variation in ranks after six years. Using data from the second year after publication as well, increases the proportion of explained variation in ranks to more than three quarters. The results also show that the impact factor of the journal in which an article was published dramatically improves the correlation between predicted and actual ranks when using just data from the year of publication and also improves the predictions based on data accrued up to one year after publication, but after that it adds little information. Finally, more than half of the articles in the top 20% in 2012 were already in the top 20% in the year of publication (2006). Based on these results, I argue that it is practical to use citation data in evaluation exercises for social sciences such as economics and political science.

The remainder of the article is structured as follows. After reviewing the existing literature on predicting future citations, I describe the data and the methods used. Then I present the results of the analysis and follow on to conclusions and discussion.

## Review of Literature on Predicting Citations

A small number of studies have computed correlations between early citations and later cumulative citations. Adams used citations to articles in the first two years after publication to predict citations in the next 3–10 years for all articles published in 1993 by UK researchers in six life and physical science fields [9]. Correlations between 1993–94 citations and 1995–2002 citations ranged from 0.94 in biochemistry and biophysics to 0.617 for optics and acoustics. Waltman *et al*. provide Pearson correlation coefficients between earlier and longer-term cumulative citations for articles published in 1999 in the fields of mathematics and biochemistry and molecular biology [10]. In mathematics the correlations with citations accumulated by the end of 2005 (equivalent to the time interval in the current study) were 0.29 at the end of 1999, 0.64 at the end of 2000, and 0.80 at the end of 2001. For biochemistry and molecular biology the equivalent correlations are 0.60, 0.85, and 0.93. Using a sample of all articles in the *Web of Science* published in 1980, Wang finds Spearman rank correlations between the partial sums of citations at 1, 3, 5, and 10 years and total citations at 31 years of 0.266, 0.754, 0.871, and 0.948, respectively [11].

Levitt and Thelwall compute rank correlations between early and cumulative citations to 2008 for all economics articles in the *Social Science Citation Index* published in 2000 with at least one UK researcher in their author list [12]. Using just citations from the year of publication they obtain a correlation of about 0.2. The correlation increases to about 0.5, 0.7, and 0.8 as the window for early citations is extended to 2001, 2002, and 2003. They also show that the impact factors of the journals the articles were published in is more predictive of future citations than the citations received in the year of publication but cumulative citations received by the end of 2001 were already more predictive of cumulative citations to 2008 than were impact factors. Similarly, using a sample of all articles in the *Web of Science* published in 1980, Bornmann *et al*. find that the impact factor and other variables (number of authors, number of references, and number of pages) can help improve predictions based on citations from the first few years after publication but have rapidly diminishing predictive power [13].

There is a larger literature on predicting citations to articles based on factors knowable at the time of publication or prior to publication but not including initial citations [14]–[19]. Additional indicators could be derived from this literature in a real world research assessment exercise. However, collecting information on authors or even the length of reference lists was prohibitively expensive for a journal article such as this and, therefore, I only use journal level information in addition to actual citations.

There are also articles that attempt to predict the number of citations that will be received by individual scientists in the future. Hirsch predicted the citations of 50 physicists at year 24 in their careers using data up till year 12 [20]. The h-index and the (square root of) total citations at year 12 both had a correlation of 0.89 with the (square root of) total citations at year 24. The h-index at year 12 and the square root of the number of citations to articles published only after year 12 at year 24 had a correlation of 0.60. Mazloumian followed this up using data from the *Web of Science* on the careers of around 150,000 scientists with non-ambiguous names [21]. He finds that an author's annual rate of total citations explains 80% of the variance in citations to existing articles in the next year and 65% of the variance in citations received in the next ten years. These percentages are somewhat more than those predicted by the author's h-index and average number of citations per article. Contrary to Hirsch [20], neither of these is a good predictor of the citations received by as yet unpublished articles.

Van Leeuwen investigates the correlation between the cumulative citations per article received by a journal for articles published in a given year in the year of their publication and each following year [22]. Economics is one of the five *Web of Science* subject categories considered. The universe of journals is split into six groups according to the number of articles published in those journals. Correlations between citations in the year of publication and cumulative citations in year two range from 0.28 to 0.89. But the correlations between cumulative citations in years two and three range from 0.94 to 0.99. This quick convergence suggests that year one and two citations are sufficient for prediction. It must be emphasized though that these correlations are at the journal level, not the article level; though the journals are sorted by size, total citations rather than impact factors are used; and cumulative citations rather than citations in each year are used. These choices will all increase the correlations relative to the alternatives.

Finally, there is research that derives more complex models of the long-term evolution of citation distributions. Wang *et al*. ask whether there is long-term predictability in citation patterns [23]. They derive a mechanistic model for the citation dynamics of individual articles, allowing them to collapse the citation histories of articles from different journals and disciplines into a single curve, indicating that all articles tend to follow the same universal temporal pattern. Their approach is to fit a model for the probability of an article being cited at time *t*:
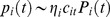
(1)where 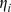 is a measure of the article's fitness, *c_it_* is the citations it has already accumulated and *P()* is a log-normal survival probability, which depends on another two parameters *µ* and *σ*. The former measures “immediacy”, governing the time for an article to reach its citation peak; and *σ* measures “longevity”, capturing the decay rate. The model can then be solved to derive a time path for *c_it_* for each individual article *i* at time *t*. This model can fit the data on article citation histories extremely nicely as many different citation patterns can be modeled. However, it seems that a considerable number of data points for each article are needed to get good estimates of the parameters. The authors make some predictions of future citations using 5 or 10 years of “training data”, but they use data with much higher than annual frequency. Still, predictions from 5 years of data do not seem that good compared to those using 10. So, this does not seem to be a practical method of generating forecasts from very narrow early citation windows.

Stringer *et al*. show that in the long run the cumulative citations to articles published in a given year in a given journal that are cited at least once converge to a lognormal distribution [24],[25]. They term the distribution when no further citations are accumulating the steady state. At any point in time, the distribution of citations to articles cited at least once follows a lognormal distribution truncated at zero. Over time, the mean increases but the standard deviation stays constant. More specifically, the steady state citations of an article *i* are given by:
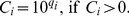
(2)where *q* is the measure of quality or popularity that explains citations. *q* follows a truncated normal distribution, truncated at zero from below with mean 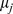 and standard deviation 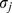 where the subscript *j* refers to a specific journal. Therefore, articles published in a specific journal share a common citation distribution. Their analysis is based on data for more than 10 million articles from the *Web of Science* database. Stringer *et al*. find that only 30 of the 2184 journals they analyze do not follow this lognormal distribution [25]. It seems that several of these are large multidisciplinary journals. These findings are useful in constructing a parametric model for forecasting cumulative citations.

## Data

I collected from the *Web of Science* all citations from 2006 to 2012 to each article published in 2006 in all journals included in the 2012 *JCR* economics and political science subject categories that had articles published and an impact factor in 2006 and remain in the index to the present as indicated by having a 5-year impact factor for 2012. This sample period should be sufficient as McCabe and Snyder find that for economics journals the annual citation rate peaks five years after publication [26]. I dropped two political science journals that had a zero impact factor in one year. Using “advanced search”, I restricted the search to the document type “articles” for items published in 2006 with results limited to 2006 to 2012. For some journals such as the *Journal of Economic Literature* or *Journal of Economic Surveys* this excludes a number of what are regular articles that are classified as “reviews” but, despite the somewhat arbitrary nature of this classification [27], I decided not to make *ad hoc* changes to the sample. It also excludes proceedings papers from journals such as the *American Economic Review* and of course, book reviews, editorials etc. I then requested a “citation report” from the database and downloaded the resulting file. In total, the sample includes 184 economics journals, which published a total of 8,715 articles in 2006 that received a cumulative total of 95,771 citations in the *Web of Science* by 2012. There are also 79 political science journals, which published a total of 2,983 articles in 2006, which received a total of 25,260 citations in the *Web of Science* by 2012. To test predictability over a longer period, I also collected data on all economics journals articles published in 1999 that meet the criteria laid out above. There are 6635 articles in this sample, which received a total of 137,064 citations by the end of 2012.

## Methods

### Software

The rank correlation and quantile analyses described below were carried out using Microsoft Excel and the regression analysis was executed using the RATS econometrics package [28].

### Simple Rank Correlation

I compute cumulative citations from 2006 to 2012 for each article as well as the partial sums for 2006, 2006–7, …, and 2006–11. I then rank all articles in each year in each discipline separately by the partial sum of citations they received up to and including that year, giving a common rank to articles with a common number of accumulated citations. I then compute the rank correlations between the 2006–2012 cumulative citations and each of the partial sums.

### Regression Models

I use a regression model to update an initial prediction based on the journal impact factor with incoming citation data as suggested by Sgroi and Oswald [7]. I use three functional forms to test the sensitivity to different specifications, though many more are obviously possible. The models are loosely based on the results of Stringer *et al*. [24]. The first regression model assumes that:
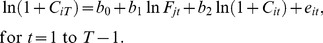
(3)Where 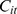 is the partial sum of citations to article *i* up till and including year *t* and 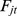 is the impact factor of the journal, *j*, in which the article was published in year *t*. Therefore, I update the impact factor as new information comes in. I add one to the citation variables in order to include articles with zero citations in the regression. I found that this model yields residuals whose absolute value is inversely related to the fitted values. An alternative model, which is often recommended for count data, is the square root transformation [29]:
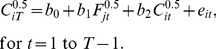
(4)


This produces less heteroscedastic residuals, though the White [30] and Breusch-Pagan [31] heteroscedasticity tests are extremely significant for all models and so I use robust standard errors clustered by journal for all regressions. Of course, if it is important to obtain more precise estimates for articles with high numbers of citations, then the heteroscedastic nature of the logarithmic model is actually advantageous because the residuals for articles with high citations are proportionally smaller. For articles with low numbers of citations, a model that explicitly takes into account the count nature of the data might be more appropriate. I fit the Poisson model to the data to see how well this works in comparison to the models that assume that the dependent variable is continuous. Again, loosely based on Stringer *et al*. [24], for all those articles with at least one citation by year *t*, I assume that the log of the mean of the distribution of the dependent variable can be modeled using:
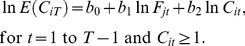
(5)


For those articles with zero citations accumulated by year *t*, I assume:
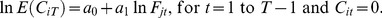
(6)


I estimate these models using the RATS command DDV with the options for count data and clustered robust standard errors. This procedure uses maximum likelihood estimation and the Newton-Raphson algorithm. More sophisticated models such as the negative binomial could also be fitted to the data, but this should not substantially affect the estimated regression coefficients [32]. As explained above, I use standard errors that take heteroskedasticity into account.

For all these models, I predict the number of citations each article will accumulate by 2012 and I then round these predictions to the nearest integer. These rounded predictions are used to rank the articles. I then compute the rank correlation coefficients for the predicted cumulative citations in 2012 for each regression estimate and the actual 2012 cumulative citations.

### Quantiles

I determine how many articles that were in the various top quantiles by cumulative citations in 2012 in each discipline were already in that top quantile in each previous year. I consider the top 20%, 10%, 5%, 2%, and 1% of articles. When articles on both sides of the nominal cutoff point have the same number of citations, I follow Bornmann *et al*. [33] by resolving these ties using the journal impact factor. I also include articles beyond the nominal cutoff point that have the same number of citations and the same journal impact factor. The number of such articles is small.

## Results

Table 1 presents the rank correlation coefficients and some additional statistics. The results for economics and political science are remarkably similar. From the fourth year on, the rate of additional citations a year is fairly constant at 20,000 for economics and 5,000 for political science. So, the citation rate seems to have settled into a steady state, though total citations are of course far from the steady state as defined by Stringer *et al*. [24]. Not surprisingly, the cumulative citations in 2010 and 2011 are highly correlated with cumulative citations in 2012. The high correlation coefficients achieved early on when only a small fraction of the 2012 cumulative citations have accumulated are more surprising. By the end of 2007 when only 8% of citations have accumulated, the correlation coefficient is 0.723 for economics and is 0.724 for political science. These imply that 53% of the variance in ranks in 2012 can be explained with less than two years on average of citation data (as the average article was published in the middle of 2006). By the end of 2008 when only 22% of citations have accumulated, the correlation coefficients are 0.880 and 0.871 implying that 77% of the variance in final ranks can already be explained. By the end of 2009 with only 40% of citations accumulated, around 90% of the variance in final ranks can be explained.

**Table 1:** Rank Correlations.

	2006	2007	2008	2009	2010	2011
Economics						
Rank Correlation Coefficient	0.359	0.729	0.880	0.949	0.977	0.993
R-Squared	0.129	0.532	0.774	0.900	0.955	0.985
Number of Cumulative Citations	1,379	7,705	20,570	37,586	56,052	75,802
Percentage of Final Citations	1.4%	8.0%	21.5%	39.5%	58.5%	79.1%
Political Science						
Rank Correlation Coefficient	0.380	0.723	0.871	0.939	0.973	0.991
R-Squared	0.144	0.523	0.758	0.882	0.947	0.983
Number of Cumulative Citations	417	2,140	5,648	10,124	14,811	19,936
Percentage of Final Citations	1.7%	8.5%	22.4%	40.1%	58.6%	78.9%

Table 2 presents regression results for the logarithmic model for economics. As expected, the coefficient of the journal impact factor declines sharply as more citation data accumulates, whereas the coefficient of the log of the partial sum of citations is fairly constant and close to unity. This implies that an article that has 1% more citations than another article already in 2006 can be expected to have 1% more cumulative citations in 2012. Given that the elasticity with respect to the partial sum of citations is unity then the intercept term is the log of the ratio of expected cumulative citations in 2012 to the partial sum of citations in the given year for an article in a journal with an impact factor of 1. The R-squared rises strongly as expected. By the end of 2008, 70% of the variation in 2012 citations can be explained by the data accumulated to date.

**Table 2:** Regression Results, Logarithmic Model: Economics.

	Constant	Log Impact Factor	Log of Partial Sum of Citations	R-Squared
2006	1.998	0.680	1.004	0.302
	(0.027)	(0.037)	(0.040)	
2007	1.580	0.438	1.008	0.500
	(0.026)	(0.026)	(0.019)	
2008	1.029	0.269	1.028	0.701
	(0.021)	(0.019)	(0.012)	
2009	0.611	0.125	1.044	0.839
	(0.015)	(0.012)	(0.008)	
2010	0.330	0.069	1.035	0.922
	(0.010)	(0.008)	(0.005)	
2011	0.142	0.031	1.016	0.969
	(0.008)	(0.004)	(0.003)	

Standard errors in parentheses.

Table 3 presents results using the square root transformation. These are similar to the results in Table 2. The intercept here is the expected square root of 2012 cumulative citations for an article with zero citations in the given year and a zero impact factor. This number is insignificantly different from zero in 2006 and in the last two years, but is significant in 2007–2009. Also, unlike the logarithmic model, the coefficient of cumulative citations declines over time. This is because the multiplier of partial citations on cumulative citations must be time-varying and declining to unity over time if the elasticity of cumulative citations with respect to partial citations is constant as we found above.

**Table 3:** Regression Results, Square Root Model: Economics.

	Constant	Square Root of Impact Factor	Square Root of Partial Sum of Citations	R-Squared
2006	0.048	2.687	1.458	0.325
	(0.137)	(0.161)	(0.081)	
2007	0.343	1.627	1.469	0.535
	(0.101)	(0.115)	(0.042)	
2008	0.159	0.917	1.422	0.734
	(0.066)	(0.062)	(0.029)	
2009	0.100	0.393	1.337	0.863
	(0.044)	(0.040)	(0.018)	
2010	0.022	0.179	1.217	0.937
	(0.027)	(0.026)	(0.009)	
2011	−0.017	0.080	1.098	0.978
	(0.013)	(0.013)	(0.004)	

Standard errors in parentheses.

The Poisson regression models in Tables 4 and 5 are comparable to the logarithmic models in Table 2 as the model is for the log of the mean of the 2012 cumulative citations. However the dependent variable for the Poisson model is simply the number of cumulative citations and, as explained above, there are separate models for articles that already received some citations and those that did not. The elasticity of the partial sum of citations rises towards unity over time and as a result the intercept needs to be larger. This suggests that articles that get some but not very many initial citations to some degree catch up with those that get more initial citations. The models for those articles without any citations in Table 5 also show a steep decline in the predictive power of the impact factor as shown by both the regression coefficient of the impact factor and the R-squared of the regression. The intercept term shows that an article that received no citations in 2006 published in a journal with an impact factor of 1 can still expect to receive a total of 10 citations by 2012. However, by 2009 we can predict that such an article will only get one citation.

**Table 4:** Regression Results, Poisson Model, Equation (5): Economics.

	Constant	Log Impact Factor	Log of Partial Sum of Citations	R-Squared
2006	2.938	0.714	0.670	0.278
	(0.039)	(0.069)	(0.079)	
2007	2.340	0.415	0.773	0.546
	(0.026)	(0.037)	(0.022)	
2008	1.595	0.259	0.861	0.775
	(0.020)	(0.019)	(0.012)	
2009	0.973	0.121	0.949	0.897
	(0.014)	(0.013)	(0.007)	
2010	0.540	0.063	0.984	0.955
	(0.011)	(0.009)	(0.005)	
2011	0.227	0.024	0.998	0.987
	(0.007)	(0.004)	(0.003)	

Standard errors in parentheses.

**Table 5:** Regression Results, Poisson Model, Equation (6): Economics.

	Constant	Log Impact Factor	R-Squared
2006	2.321	0.859	0.185
	(0.027)	(0.044)	
2007	1.799	0.608	0.124
	(0.030)	(0.039)	
2008	1.027	0.459	0.059
	(0.034)	(0.048)	
2009	0.250	0.290	0.024
	(0.048)	(0.059)	
2010	−0.548	0.286	0.014
	(0.065)	(0.077)	
2011	−0.537	0.276	0.014
	(0.067)	(0.068)	

Standard errors in parentheses.

Tables 6 to 9 present the regression results for political science. These are similar to those for economics, though, of course, the sample sizes are smaller and the standard errors larger. Comparing Table 6 to Table 2, the main difference is that the effect of the impact factor declines more slowly in political science. There is a similar pattern when using the square root model (Table 7 and Table 3). The greatest differences are for the Poisson models (Tables 8 and 4 and Tables 9 and 5). The R-squared in 2006 for equation (5) for political science is almost twice as large as that for economics. Articles that already got at least one citation in the first year in political science are more clearly destined to be outstanding. The coefficient of the log of the partial sum of citations is also much larger in 2006 for political science than for economics. Here there is no catch-up effect for slow-starting articles. There is a catch-up effect in following years, but it is weaker than in economics. The results for equation (6) are even more different. For political science, the explanatory power of the impact factor for articles that did not yet receive any citations actually rises until 2008 and the size of the effect remains stronger than in economics though the R-squared eventually falls to a similar level in 2011. It seems that, despite the lack of a catch-up effect among articles that already received some citations in 2006, there are some high quality articles published in the higher impact journals that are slow to receive citations. This effect is much weaker in economics.

**Table 6:** Regression Results, Logarithmic Model: Political Science.

	Constant	Log Impact Factor	Log of Partial Sum of Citations	R-Squared
2006	1.948	0.710	1.060	0.397
	(0.064)	(0.093)	(0.071)	
2007	1.525	0.597	1.069	0.594
	(0.053)	(0.075)	(0.034)	
2008	0.981	0.385	1.045	0.765
	(0.038)	(0.046)	(0.022)	
2009	0.538	0.211	1.068	0.860
	(0.056)	(0.023)	(0.029)	
2010	0.289	0.096	1.057	0.930
	(0.043)	(0.011)	(0.020)	
2011	0.106	0.032	1.034	0.974
	(0.024)	(0.007)	(0.011)	

Standard errors in parentheses.

**Table 7:** Regression Results, Square Root Model: Political Science.

	Constant	Square Root of Impact Factor	Square Root of Partial Sum of Citations	R-Squared
2006	−0.216	2.851	1.606	0.361
	(0.349)	(0.419)	(0.224)	
2007	−0.304	2.201	1.510	0.590
	(0.237)	(0.267)	(0.074)	
2008	−0.203	1.217	1.418	0.770
	(0.127)	(0.145)	(0.047)	
2009	−0.141	0.604	1.325	0.873
	(0.081)	(0.080)	(0.031)	
2010	−0.071	0.287	1.208	0.941
	(0.037)	(0.041)	(0.017)	
2011	−0.018	0.066	1.105	0.980
	(0.015)	(0.022)	(0.008)	

Standard errors in parentheses.

**Table 8:** Regression Results, Poisson Model, Equation (5): Political Science.

	Constant	Log Impact Factor	Log of Partial Sum of Citations	R-Squared
2006	2.777	0.687	1.164	0.498
	(0.090)	(0.089)	(0.217)	
2007	2.283	0.489	0.845	0.584
	(0.060)	(0.065)	(0.054)	
2008	1.510	0.268	0.908	0.813
	(0.051)	(0.046)	(0.046)	
2009	0.908	0.160	0.971	0.904
	(0.042)	(0.025)	(0.023)	
2010	0.528	0.080	0.991	0.959
	(0.032)	(0.015)	(0.014)	
2011	0.215	0.028	1.005	0.990
	(0.015)	(0.007)	(0.006)	

Standard errors in parentheses.

**Table 9:** Regression Results, Poisson Model, Equation (6): Political Science.

	Constant	Log Impact Factor	R-Squared
2006	2.286	0.850	0.153
	(0.070)	(0.122)	
2007	1.742	0.893	0.169
	(0.066)	(0.135)	
2008	0.961	0.930	0.179
	(0.069)	(0.145)	
2009	0.129	0.894	0.096
	(0.013)	(0.021)	
2010	−0.726	0.811	0.067
	(0.189)	(0.253)	
2011	−1.996	0.600	0.013
	(0.378)	(0.179)	

Standard errors in parentheses.

Table 10 presents the correlations between the predicted ranks in 2012 using data up to the year given and the actual ranks. The correlations are similar to those in Table 1 with the exception of the correlation for 2006. The results are remarkably similar across functional forms and disciplines despite the differences in the regression results documented above. Comparing Table 10 with Table 1, the R-Squared more than doubles for 2006 when the impact factor data is also used. However, in 2007 the additional information only adds 5–6% to the explained variance. By 2008 the additional explanatory power is only 2%. So while impact factors are useful in predicting future citations in the first year or two after publication, they add little explanatory power after that. An obvious criticism of the regression analysis in this article is that if we want to carry out an evaluation exercise of a set of articles not long after they are published we will not have the information on future cumulative citations, which was used to estimate these regression models. But because the explanatory power of the impact factor declines rapidly, just using the rank analysis in Table 1 will be an adequate predictor of the future ranks of articles after a couple of years of information are acquired. It is not necessary to fit a model to data as we have done in Table 10 in order to generate good predictions. Even if a model is used, the exact functional form and parameter values do not seem to be important. Levitt and Thelwall show that rank correlations with future cumulative citations are not very sensitive to the weightings used for early citations and journal impact factors in the predictor [12].

**Table 10:** Predicted Rank Correlations.

	2006	2007	2008	2009	2010	2011
Economics						
Logarithmic						
Rank Correlation Coefficient	0.543	0.765	0.890	0.952	0.978	0.993
R-Squared	0.295	0.586	0.792	0.906	0.957	0.985
Square Root						
Rank Correlation Coefficient	0.547	0.744	0.879	0.952	0.975	0.992
R-Squared	0.300	0.553	0.773	0.906	0.952	0.983
Poisson						
Rank Correlation Coefficient	0.554	0.762	0.886	0.949	0.977	0.991
R-Squared	0.307	0.580	0.786	0.901	0.954	0.983
Political Science						
Logarithmic						
Rank Correlation Coefficient	0.540	0.765	0.879	0.941	0.974	0.991
R-Squared	0.291	0.585	0.772	0.886	0.949	0.983
Square Root						
Rank Correlation Coefficient	0.528	0.753	0.873	0.939	0.973	0.991
R-Squared	0.279	0.567	0.762	0.881	0.946	0.982
Poisson						
Rank Correlation Coefficient	0.530	0.764	0.881	0.941	0.973	0.990
R-Squared	0.281	0.584	0.776	0.886	0.947	0.979

Table 11 shows what fraction of the articles in each indicated quantile was already in that quantile in earlier years. The fraction of the top 20% of articles in 2012 that were already in this quantile in 2006 is 51% for economics and this increases to 60% by the end of 2007 and 74% by the end of 2008. It is more difficult to predict which articles would be in the higher quantiles using data from the first two years. This difference in predictability diminishes as citations accumulate. By 2008, 74% of the top 20% of articles and 70% of the top 1% of articles can be predicted. Therefore, this seems a fairly useful tool for assessing which departments, for example, have publications in the top 20% only 2 to 3 years after publication.

**Table 11:** Quantile Persistence: Economics.

Top	2006	2007	2008	2009	2010	2011
20%	0.510	0.600	0.742	0.818	0.881	0.931
10%	0.356	0.553	0.708	0.800	0.864	0.917
5%	0.328	0.530	0.665	0.810	0.881	0.933
2%	0.259	0.546	0.667	0.799	0.868	0.931
1%	0.172	0.471	0.701	0.793	0.874	0.931

Again, the results for political science are similar to those for economics (Table 12), though, at least in this sample, it is easier to predict which articles will be higher ranked with just the first year of data than it is for economics.

**Table 12:** Quantile Persistence: Political Science.

Top	2006	2007	2008	2009	2010	2011
20%	0.506	0.643	0.750	0.826	0.883	0.928
10%	0.342	0.587	0.691	0.768	0.842	0.903
5%	0.275	0.537	0.651	0.752	0.839	0.926
2%	0.300	0.550	0.733	0.783	0.917	0.917
1%	0.333	0.433	0.733	0.733	0.867	0.933

## Discussion and Conclusions

The desire to rank articles, researchers, and institutions [3] is not likely to diminish, as ranking behavior is inherent in humans [34] and, of course, other primates [35]. The question is how to carry out a ranking in an accurate and cost-effective way. I find in this article that it is possible to forecast the future citations rank of journal articles in two social science disciplines fairly well using data available from citation databases within the first few years following publication. I also found that the journal impact factor is quite useful in predicting future citations and rank in the first two years following publication. However, its usefulness drops steeply as more actual citations data accumulates. It more than doubles the explained variance in rank in 2012 using just data from 2006. But by the third year it only adds 2% to the explained variation. This means that ranking by accumulated citations in the first few years following publication should be sufficient to predict the future citation ranking of journal articles in these disciplines. If predictions are required using only the first year of citations, then impact factors and other variables can also be used [13].

To test the robustness of the analysis, I also analyze the citations received by economics journal articles published in 1999 (Table 13). This allows us to observe the accumulation of citations over twice as many years as the main analysis reported in this article. Comparing Tables 1 and 13, the correlations between the partial sums of citations and 2005 cumulative citations are very similar for the two samples. A slightly larger fraction of the final citations accumulated in the first couple of years in the 1999 sample. Comparing the correlations with 2012 cumulative citations and the correlations with 2005 cumulative citations in Table 13, it takes more time to generate a similar correlation with 2012 cumulative citations than it does with 2005 cumulative citations. But a smaller fraction of final citations is needed to generate the same magnitude of correlation. We can still explain more than half the final variation in ranks using data from the first three years.

**Table 13:** Rank Correlations for Economics Articles Published in 1999.

		2005			2012		
	Cumulative Citations	Correlation with Final Citations	R-Squared	Fraction of Final Citations	Correlation with Final Citations	R-Squared	Fraction of Final Citations
1999	897	0.398	0.159	0.020	0.326	0.106	0.007
2000	4215	0.698	0.488	0.094	0.597	0.357	0.031
2001	10147	0.856	0.732	0.225	0.752	0.565	0.074
2002	17585	0.932	0.869	0.391	0.828	0.686	0.128
2003	26105	0.971	0.942	0.580	0.874	0.764	0.190
2004	35154	0.991	0.983	0.781	0.903	0.816	0.256
2005	45011				0.927	0.860	0.328
2006	55904				0.945	0.894	0.408
2007	67833				0.961	0.924	0.495
2008	81328				0.974	0.949	0.593
2009	95501				0.984	0.968	0.697
2010	109508				0.990	0.981	0.799
2011	123587				0.995	0.991	0.902
2012	157008						

Comparing my results with previous similar studies, I find some similarities and some differences. The correlations I find in my global sample between early citations and final citations both 7 and 14 years from publication are higher than those that Levitt and Thelwall find for British economics articles [12]. In common with both Levitt and Thelwall [12] and Bornmann *et al*. (2014) [13], I find that journal impact factors have a rapidly diminishing contribution to helping predict future citations. Comparing this study to Wang [11], the rank correlations between partial sums of citations and cumulative citations at 14 years are similar to the correlations that he finds for citations at 31 years in all disciplines. Comparing my results to those for the specific disciplines analyzed by Waltman *et al*. [10], my findings for economics and political science show higher predictability than they find for mathematics, but, not surprisingly, less than they find for biochemistry and molecular biology. Comparing my results to those of Adams [9], I find correlations of 0.692 between 2006–7 and 2008–12 citations for economics and 0.718 for political science, which are comparable to his results for the physical sciences.

My results suggest that citation analysis could be used more widely in research assessment exercises in the social sciences than it currently is. Existing research finds strong correlations between the rankings produced by UK research assessment exercises and bibliometric analyses for several specific humanities and social science disciplines including economics [36]–[39]. Research does show that peer review at journals has predictive validity for the citations that will be received by accepted papers compared to those received by rejected papers. However, evidence for the predictive validity of peer review of grant and fellowship applications is more mixed [40]. Therefore, further research is warranted on use of citation analysis to rank academic departments or universities in research assessment exercises.
